# Supplementary material for: Inside out: transforming images of lab-grown plants for machine learning applications in agriculture
Source: Front Artif Intell. 2023 Jul 6;6:1200977. doi: 10.3389/frai.2023.1200977 (PMC10358354; doi:10.3389/frai.2023.1200977)
Supplement: Supplementary file 1 [file Data_Sheet_1.pdf]

Inside Out: Transforming Images of Lab-Grown Plants for Machine Learning Applications in Agriculture – Supplementary Material

Alexander E. Krosney [1,2], **Parsa Sotoodeh** [3], **Christopher J. Henry** [3,*], **Michael A. Beck** [3] **and Christopher P. Bidinosti** [2,3]

[1] *University of Manitoba, Department of Computer Science, Winnipeg, Manitoba, Canada*
[2] *University of Winnipeg, Department of Physics, Winnipeg, Manitoba, Canada*
[3] *University of Winnipeg, Department of Applied Computer Science, Winnipeg, Manitoba, Canada*

Correspondence*:
Christopher J. Henry
ch.henry@uwinnipeg.ca

This is supplementary material for the Article *Inside Out: Transforming Images of Lab-Grown Plants for Machine Learning Applications in Agriculture* with DOI 10.3389/frai.2023.1200977. This document contains the two appendices below for this article.

APPENDIX 1 IMPROVED INDOOR BOUNDING BOX ALGORITHM

Here we present an algorithm used to obtain tighter bounding boxes for our multi-plant lab images. The algorithm functions by mapping all sub-regions of a multi-plant image to the correct plants. For the purpose of this paper, we will refer to these regions as fragments.

Initial, loose bounding boxes are immediately determined through geometric calculations relating the plant position in the scene and the camera position/angle. These initial bounding boxes include an additional tolerance to help ensure that the plants are fully contained within the box, resulting in a conservative estimate for the plant position within a given image. The loose bounding boxes are readily available in the indoor plant database when downloading the multi-plant images. An example of an indoor multi-plant image with the original bounding boxes is given by Figure 19.

The first step is to remove the image background, here we convert a BGR image to CIELAB color space. The benefit to operating in CIELAB space is in the a- and b-channels of the image. The a-channel has a low magnitude for green pixels, and large for red. Similarly, the b-channel has a low magnitude for blue pixels, and large for yellow. We create an overall image mask through addition of a- and b-channel masks, where we set any pixel with a-value greater than `a_cutoff` to zero and any pixel with b-value less than `b_cutoff` to zero. The addition of the two masks results in a value of 0 anywhere the a-channel of a pixel is greater than `a_cutoff` and the b-channel is less than `b_cutoff` and a value of 255 for all other pixels in the image. In general, we assign the values `a_cutoff` $= 130$ and `b_cutoff` $= 95$. A border around the image is also constructed so that plants that reach the outside of the image are correctly labelled as fragments in the proceeding step. Algorithm 2 provides the code, written in NumPy and OpenCV, used to construct the image mask.

Next, all fragments in the masked image are found. Image opening (equivalent to erosion, followed by dilation) is used to remove small fragments in the image that would otherwise be difficult to assign to or may not belong to any plant. Then, any fragments with size below `size_cutoff` (typically set to 200 px) are removed. Algorithm 3 provides the code for this operation.

We now possess several image fragments with a unique label and must associate each to a plant in the original image. For each fragment $f \in F$, the centre of mass $c(f) = (x_f, y_f)$ is evaluated where x_f and y_f are the x- and y-coordinates of the fragment, respectively. Additionally, for each plant $p \in P$, the centre of the plant $c(p) = (x_p, y_p)$ is found using the image metadata. We initialize a distance matrix $D \in \mathrm{Mat}(|\mathrm{P}| \times |\mathrm{F}|)$ where $D := (d(p, f))$ and $d(p, f)$ is the Euclidean distance between the centre of the plant p and the centre of mass of the fragment f.

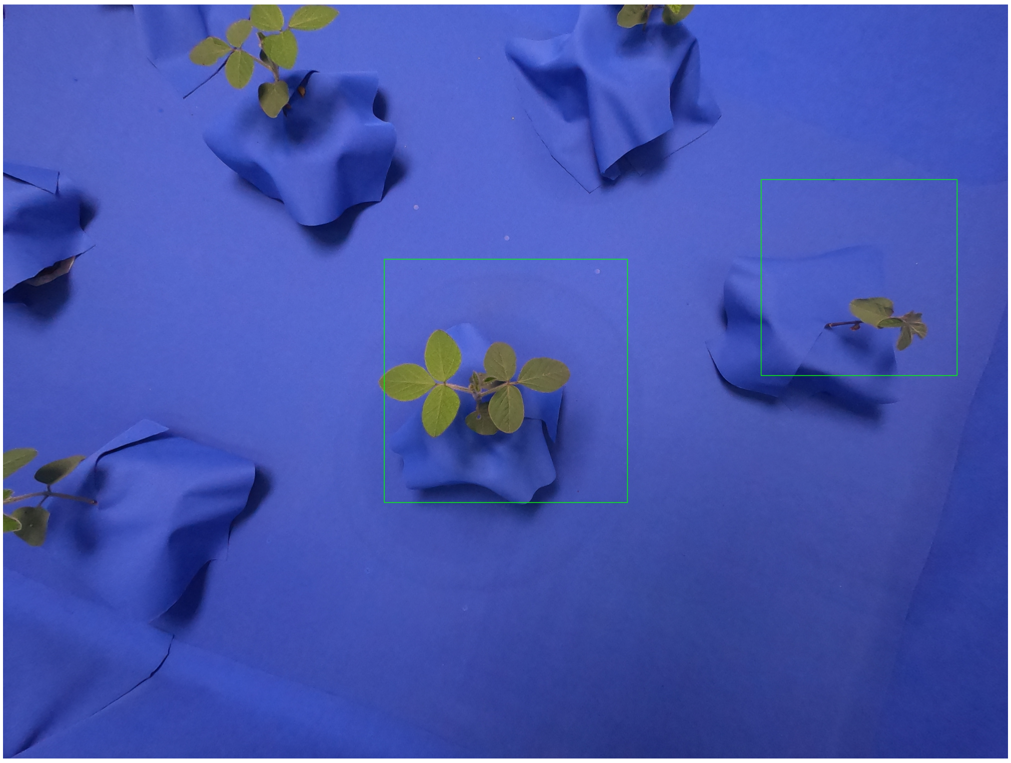

Figure 19. Indoor multi-plant image with the original bounding boxes shown in green. A conservative estimate of the plant position leads to loose bounding boxes for each plant. Plants that are overlapping or are too close to the image border do not receive bounding boxes.

Algorithm 2 Algorithm for removing the multi-plant image background

```
import cv2
import numpy as np

# Input: master = original image read as 3-dimensional array

# convert master into LAB color space and extract b-channel, a-channel
lab = cv2.cvtColor(master, cv2.COLOR_BGR2LAB)
a_channel = np.array(lab[ :, :, 1])
b_channel = np.array(lab[ :, :, 2])

# masking
_, b_mask = cv2.threshold(b_channel, b_cutoff, 128, cv2.THRESH_BINARY)
_, a_mask = cv2.threshold(a_channel, a_cutoff, 127, cv2.THRESH_BINARY_INV)
mask = (a_mask + b_mask)
mask[mask < 129] = 0

# introduce 1px frame around interior
mask[:, 0] = 0
mask[:, mask.shape[1] - 1] = 0
mask[0, :] = 0
mask[mask.shape[0] - 1, :] = 0
```

Algorithm 3 Algorithm for generating fragments from mask

```
import cv2
import numpy as np
from scipy.ndimage import measurements

# open mask for fast removal of small fragments
kernel = np.ones((k_size, k_size), np.uint8)
thresholded = cv2.morphologyEx(mask, cv2.MORPH_OPEN, kernel)

# label fragments
labels, n_fragments = measurements.label(thresholded)
thresholded_copy = thresholded.copy()

# remove small fragments, 0 is the background
for frag_number in range(1, n_fragments+1):
    if measurements.sum(thresholded, labels, frag_number) < size_cutoff*255:
        slices = find_objects(labels == frag_number)[0]
        thresholded_copy[slices[0].start: slices[0].stop, slices[1].start: slices[1].stop] = 0

# relabel fragments
labels, n_fragments = measurements.label(thresholded_copy)
```

Finally, we reduce extraneous fragments by finding any with an unreasonable distance to a plant in the original image. To achieve this, the radius of the plant in the image $r(p)$ is estimated by calculating the width of the bounding box from the original metadata. Equivalently, the bounding box height could be used to calculate the radius since the original bounding boxes are all square. All fragments that satisfy $D_{p,f} > r(p) \cdot T_1$ are removed, where T_1 is a distance threshold multiplier that is typically assigned the value $T_1 = 1.6$. From the remaining valid fragments, the one with the smallest Euclidean distance is assigned to the plant. The output of Figure 19 after the bounding box tightening algorithm is given in Figure 20.

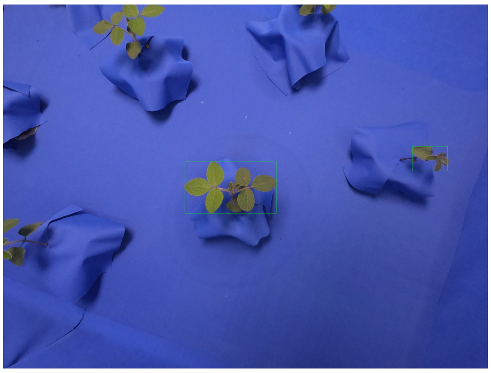

Figure 20. Indoor multi-plant image with the improved bounding boxes shown in green. The bounding boxes are significantly tighter than the originals.

APPENDIX 2 ADDITIONAL COLOR-CORRECTED COMPOSITE TRANSLATION RESULTS

This section contains image translation results for generators trained to translate color-corrected composite images of canola, oat, and wheat. The datasets used for generator training are similar to the *Color-Corrected Composites 1* dataset of Table 1. Twenty additional color-corrected composite photos of each plant from the same age range unseen during the training process compose the distribution V for qualitative evaluation of the models. Dataset parameters for this section are listed in Table 4.

Dataset Name	N_x	N_y	Species	Age (days)	$N_{\text{backgrounds}}$	S_{min}	S_{max}	Figure
Color-Corrected Composites 4	64	64	Canola	10-40	32	0.50	0.85	21
Color-Corrected Composites 5	64	64	Oat	0-365	32	0.50	0.85	22
Color-Corrected Composites 6	64	64	Wheat	0-365	32	0.50	0.85	23

Table 4. Parameters for each additional training dataset.

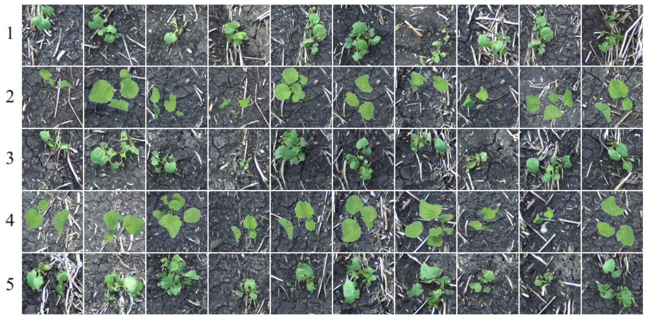

Figure 21. Canola images sampled from the distribution Y (1), images sampled from the distribution X (2), translated images $G(\boldsymbol{x})$ (3), images sampled from the distribution V (4), and translated images $G(\boldsymbol{v})$ (5).

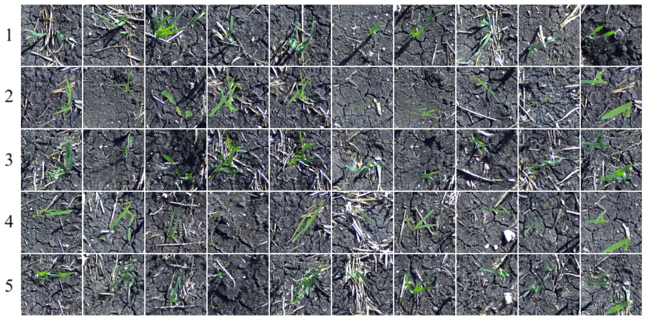

Figure 22. Oat images sampled from the distribution Y (1), images sampled from the distribution X (2), translated images $G(\boldsymbol{x})$ (3), images sampled from the distribution V (4), and translated images $G(\boldsymbol{v})$ (5).

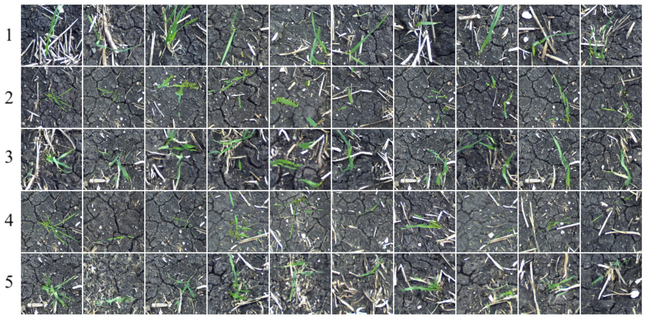

Figure 23. Wheat images sampled from the distribution Y (1), images sampled from the distribution X (2), translated images $G(\boldsymbol{x})$ (3), images sampled from the distribution V (4), and translated images $G(\boldsymbol{v})$ (5).
